# Distinct morphologies of arterial waveforms reveal preload‐, contractility‐, and afterload‐deficient hemodynamic instability: An in silico simulation study

**DOI:** 10.14814/phy2.15242

**Published:** 2022-04-12

**Authors:** Marijn P. Mulder, Michael Broomé, Dirk W. Donker, Berend E. Westerhof

**Affiliations:** ^1^ Cardiovascular and Respiratory Physiology TechMed Centre University of Twente Enschede The Netherlands; ^2^ Anesthesia and Intensive Care Department of Physiology and Pharmacology Karolinska Institute Stockholm Sweden; ^3^ 59562 ECMO Department Karolinska University Hospital Stockholm Sweden; ^4^ Intensive Care Center University Medical Center Utrecht Utrecht The Netherlands; ^5^ Department of Pulmonary Medicine Amsterdam Cardiovascular Sciences Amsterdam UMC Vrije Universiteit Amsterdam Amsterdam The Netherlands

**Keywords:** arterial waveform, cardiovascular simulation, hemodynamic instability, principal component analysis, shock

## Abstract

Hemodynamic instability is frequently present in critically ill patients, primarily caused by a decreased preload, contractility, and/or afterload. We hypothesized that peripheral arterial blood pressure waveforms allow to differentiate between these underlying causes. In this in‐silico experimental study, a computational cardiovascular model was used to simulate hemodynamic instability by decreasing blood volume, left ventricular contractility or systemic vascular resistance, and additionally adaptive and compensatory mechanisms. From the arterial pressure waveforms, 45 features describing the morphology were discerned and a sensitivity analysis and principal component analysis were performed, to quantitatively investigate their discriminative power. During hemodynamic instability, the arterial waveform morphology changed distinctively, for example, the slope of the systolic upstroke having a sensitivity of 2.02 for reduced preload, 0.80 for reduced contractility, and −0.02 for reduced afterload. It was possible to differentiate between the three underlying causes based on the derived features, as demonstrated by the first two principal components explaining 99% of the variance in waveforms. The features with a high correlation coefficient (>0.25) to these principal components are describing the systolic up‐ and downstroke, and the anacrotic and dicrotic notches of the waveforms. In this study, characteristic peripheral arterial waveform morphologies were identified that allow differentiation between deficits in preload, contractility, and afterload causing hemodynamic instability. These findings are confined to an in silico simulation and warrant further experimental and clinical research in order to prove clinical usability in daily practice.

## Graphical abstract

In this in‐silico simulation study, characteristic peripheral arterial pressure waveform morphologies were identified that allow differentiation between deficits in preload, contractility, and afterload causing hemodynamic instability.

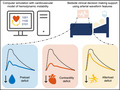

## 1 INTRODUCTION

Hemodynamic (HD) instability is a major cause of admission to the intensive care unit (ICU) and about one third of all ICU patients are in an overt circulatory shock (Cecconi et al., 2014). The causes of HD instability can broadly be divided into deficits in preload, contractility, and afterload, or a combination (Teboul et al., 2016). This all may cumulate into decreased organ perfusion and potentially severe and progressive multiorgan failure, the latter being associated with high mortality (Cecconi et al., 2014). Therefore, effective management of HD instability and shock is a major focus in the ICU.

In order to manage HD instability in the best way, it is of importance to determine the etiology and the pathophysiological mechanisms involved. Often the exact underlying cause, or the combination of causes, cannot directly be unraveled. Therefore, HD management of these patients is frequently based on a pragmatic clinical approach, adhering as much as possible to standard hemodynamic monitoring and current guidelines. Ideally, a choice for fluid resuscitation and eventual concomitant use of vasopressors or inotropic medication should be based on individual pathophysiological insights. This leads to more personalized and rational HD management, which may reduce ICU stay (Goepfert et al., 2013).

A universal and attractive element of individual clinical HD monitoring is the peripheral arterial waveform, since it provides information on both cardiac and vascular function (Esper & Pinsky, 2014). Arterial lines are routinely used in the ICU and therefore high‐frequency sampled arterial waveform measurements are easily accessible. In current clinical practice, these arterial blood pressure data are mainly used to record systolic (SBP), diastolic (DBP), pulse (PP), and mean arterial pressure (MAP), but the diagnostic potential of the arterial waveform has not been fully exploited.

So far, HD management guided by these invasive blood pressure data focuses on pulse contour analysis to estimate cardiac output (CO) and pulse pressure variation (PPV), as measure of fluid responsiveness (Teboul et al., 2016). The use of pulse contour analysis is limited, since it can only be applied under strict conditions, is not reliable in unstable patients and does not differentiate well between underlying pathophysiological causes of HD instability (Monnet et al., 2016; Teboul et al., 2016). In hypertension research, arterial waveform analysis has contributed to better quantification on the afterload since the 1980s (Chirinos & Segers, 2010; Murgo et al., 1980). Currently, waveform analysis for classification of blood loss or hypovolemia (Convertino et al., 2016; van der Ster et al., 2018a, 2018b, 2021) and prediction of hypotension is gaining momentum (Hatib et al., 2018; Wijnberge et al., 2020).

We hypothesize that clinically relevant changes in preload, contractility and afterload will be reflected by distinct peripheral arterial waveform morphologies, allowing differentiation between the various causes in a strictly controlled in silico analysis. We aim primarily to provide a tool for clinical research phenotyping HD instability based on arterial waveform analysis and eventually to improve HD management of individual patients.

## 2 MATERIALS AND METHODS

### 2.1 Cardiovascular simulations

To study adult human hemodynamics, the previously described ‘Aplysia CardioVascular Lab’ (version 9.5.7.0, 2021, Aplysia Medical AB, Stockholm, Sweden) (Broomé et al., 2013; Broomé & Donker, 2016; Lindfors et al., 2017; Donker et al., 2019; Maksuti et al., 2019) was used. This is a real‐time closed‐loop lumped parameter model based on an electrical analog of the human cardiovascular system with resistances, inductances, and capacitances. It consists of 32 zero‐dimensional compartments; solely the pressure in the peripheral artery compartment is taken into account, since this relates the most to invasively measured blood pressure with arterial lines in clinical practice. This simulator deploys realistic time‐varying elastance curves to describe the function of the heart chambers and provides detailed peripheral arterial pressure waveforms.

As a starting point, a 60‐year‐old adult was selected, which reflects the mean age of the ICU population (Garland et al., 2013), defined in Aplysia as default with a set of input variables (Table 1) and designated as hemodynamically stable. Ventilation was turned off. Next, for the design of the simulation scenarios carried out in this study, the cardiovascular concept of contractility was simplified by only taking the left ventricle into account. Aortic compliance and other potentially influencing factors were neglected. Similarly, afterload was interpreted solely as vasomotor tone and preload substituted with the total effective circulating volume. To simulate HD instability caused by reduction of preload, contractility or afterload, the input variables blood volume (BV), left ventricular contractility (LVC), and systemic vascular resistance (SVR) were decreased, respectively. Simulations were carried out with stepwise reductions of the respective input variables until a DBP or PP <20 mmHg, or MAP <30 mmHg was reached. LVC and SVR were both decreased stepwise by 5% each time. Since the model is more sensitive to reductions in BV, a step size of 2% was chosen for this input variable.

**TABLE 1:** Settings in Aplysia of default male adult cases with normal physiology and different age

Variable	20 years	40 years	60 years	80 years
Height [cm]	170	170	170	170
Weight [kg]	60	75	80	70
Body surface area [m^2^]	1.683	1.882	1.944	1.818
Body mass index [kg/m^2^]	20.8	26.0	27.7	24.2
Heart rate [/min]	74	71	65	72
Blood volume [ml]	4767	5958	6355	5561
Left ventricular contractility [mmHg/ml]	3.02	2.71	2.62	2.80
Left ventricular stiffness [mmHg/ml]	0.032	0.029	0.028	0.030
Right ventricular contractility [mmHg/ml]	0.77	0.67	0.65	0.70
Right ventricular stiffness [mmHg/ml]	0.016	0.014	0.014	0.015
Systemic vascular resistance [mmHg*s/ml]	1.28	1.13	1.09	1.18
Pulmonary vascular resistance [mmHg*s/ml]	0.141	0.121	0.116	0.127
Venous compliance [ml/mmHg]	173	140	106	73
Systemic arterial stiffness [mmHg/ml]	0.44	0.57	0.76	1.07
Pulmonary arterial stiffness [mmHg/ml]	0.24	0.30	0.41	0.58
Youngs modulus [mmHg]	1.694	2.361	3.250	4.361

To study the effects of age and individual body size differences on waveform morphology, the same interventions as outlined above were repeated to create hemodynamic instability, with default cases of adult patients of 20, 40, and 80 years old as defined in Aplysia (Table 1). The only variable changed with age is Youngs modulus of the blood vessels, which is linearly scaled to obtain realistic aortic pulse wave velocity and blood pressures comparable to literature data (Franklin et al., 1997; Maksuti et al., 2016). Since body surface area (BSA) varies between the different default adult cases, heart rate (HR), blood volume and cardiac and vascular properties are scaled accordingly (Table 1) (Neilan et al., 2008, 2009).

In order to systematically explore the effects of baroreflex compensatory mechanisms on the arterial waveform during HD instability, additional scenarios were simulated. Sympathetic activation may lead to increased heart rate, cardiac contractility, vascular resistance, and/or decreased venous compliance (VC) to restore blood pressure and flow. As a starting point, a hemodynamically unstable 60‐year‐old adult with a MAP of 60 mmHg was chosen, either caused by a reduced preload, contractility, or afterload. Next, per underlying cause the input variables HR, LVC, and SVR were gradually increased, and VC decreased, separately until recovery from hemodynamic instability. Recovery was defined as a MAP of 70 mmHg; when blood pressure did not increase to this level, a maximum increase or reduction of 100% of the baroreflex variable was used. For obvious reasons, LVC was not increased in the scenario of reduced contractility, since this simply removes the underlying cause, and the same holds for SVR in the reduced afterload scenario and VC in the reduced preload case.

Additionally, the effect of spontaneous ventilation on the arterial waveforms was studied by performing the same initial simulations of a 60‐year‐old adult with hemodynamic instability, but now breathing with a respiratory rate of 11/min and a tidal volume of 0.5 liter.

### 2.2 Arterial waveform analysis

**FIGURE 1:**
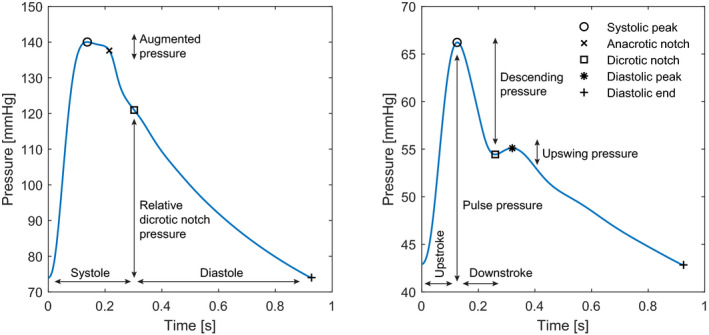
Two arterial waveforms with the systolic peak, dicrotic notch, and diastolic end. In the left panel, the division of the arterial waveform in systole and diastole by the dicrotic notch is depicted. The relative dicrotic notch pressure is indicated here. A C‐type anacrotic notch is visible in the left panel, indicated together with the augmented pressure. In the right panel, the division between systolic upstroke and downstroke is shown. Three relative pressures are indicated: pulse pressure, descending pressure, and diastolic upswing pressure

After changing an input variable, the output data were captured only after the model had reached an equilibrium in CO and MAP. Since the model consists of multiple time‐dependent equations, it could take a couple of heartbeats before the full effect of the intervention was seen and a ‘stable’ situation was obtained. The peripheral arterial blood pressure (in mmHg) and flow waveforms (in ml/s) generated by Aplysia with a sample frequency of 400 Hz were analyzed using MATLAB^®^ software (version R2021B, The Mathworks, Inc., Natick, MA, USA). First, a single heartbeat from the pressure waveform was selected and five fiducial points to specify waveform morphology were detected (Figure 1).

The systolic peak was defined as the maximum pressure during a heartbeat, diastolic end as the minimum pressure after the systolic peak. The dicrotic notch marks the end of systole and the beginning of the diastolic phase (Esper & Pinsky, 2014); detection was based on a maximum in the first derivative or a minimum in the second derivative of the signal (Singh & Sunkaria, 2017). The anacrotic notch is a result of wave reflection and can be present before the systolic peak (A‐type) or after the systolic peak (C‐type) (Murgo et al., 1980; Segers et al., 2007). The A‐ and C‐type anacrotic notch are defined by a shoulder point, respectively a maximum or minimum in the third derivative, or an inflection point, maximum of the second derivative of the signal (Segers et al., 2007). An example of a C‐type anacrotic notch can be found in the left panel of Figure 1. When a dicrotic notch could not be identified, it was set at 1/3th of the heartbeat, and the anacrotic notch was set at the systolic peak when no infection or shoulder point could be defined.

In total, 45 features per heartbeat were defined to describe the waveform morphology, for an overview of all definitions and formulas (see Appendix A‐Table A1). For each of the fiducial points the absolute pressure was calculated. Also relative pressures between points were defined; indicated in Figure 1. Indices were obtained by dividing the relative pressures over the pulse pressure (Nürnberger et al., 2002).

Using the fiducial points the waveform can be divided into different parts in time, representing different phases of the cardiac cycle: systole, with an upstroke and downstroke, and diastole (Esper & Pinsky, 2014) (Figure 1). For each part, the duration was calculated, the average slope of the curve and area under the waveform. Also, relative areas were defined, by subtracting the part below diastolic pressure, and the myocardial oxygen supply/demand ratio was calculated by dividing diastolic area over the systolic area (Buckberg et al., 1972).

Furthermore, the Liljestrand and Zander formula was used with a calibration factor of 3.5 to estimate stroke volume and cardiac output (Koenig et al., 2015). Lastly, the pressure waveform was separated into the forward and reflected wave by estimating the characteristic impedance in the frequency domain (5–15 Hz), using the peripheral flow waveform output from Aplysia (Westerhof et al., 1972; Qureshi et al., 2018). The exact and more elaborate description of these estimations can be found in Appendix A‐Table A1. From the forward and reflected wave absolute and relative peak pressures, time to peak and area under the waveform were defined.

### 2.3 Feature analysis

To investigate whether and to what extent the calculated arterial waveform features are sensitive to changes in input variables, a one‐at‐the‐time sensitivity analysis was performed. This sensitivity analysis could also be used to check if features change differently for the three HD instability causes, respectively. The scenarios of a 10% and 30% decrease in the variables BV, LVC, and SVR and an additional simulation of 10% increase of the 60‐year‐old adult were used. The sensitivity was calculated by dividing the percental change in feature over the percentual change in the input variable.

A principal component analysis (PCA) was performed to investigate whether it is possible to distinguish the three main underlying causes of hemodynamic instability based on arterial waveforms. The raw waveforms of the 60‐year‐old adult with stepwise decrease of BV, LVC, and SVR were used as input for the PCA. The correlation coefficient plotted against time shows which phases of the waveform are most important for differentiation. Next, the calculated arterial waveform features were used as input for the PCA. The correlation coefficient then shows the most important features. The same PCA was performed with all simulated adult cases with different ages and stepwise decrease of BV, LVC, and SVR.

## 3 RESULTS

### 3.1 Simulated arterial waveforms

**FIGURE 2:**
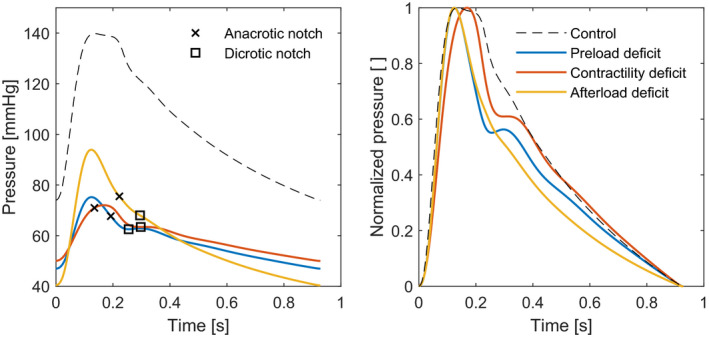
Arterial waveforms of a simulated 60‐year‐old adult with normal physiology (black), reduced blood volume (blue, −26%), reduced left ventricular contractility (red, −85%) and reduced systemic vascular resistance (yellow, −60%). The examples illustrating the reduced situations were selected based on a mean arterial pressure of around 60 mmHg to facilitate comparison. In the left panel absolute waveforms with detected notches are displayed, showing a reduction in pressure and distinct change in morphology when the three variables are reduced. In the right panel the same waveforms are displayed, but now normalized, ranging from 0 to 1 in pressure. The differences in morphology can be seen more clearly here: reduction of the three variables changes the waveform shape differently

In total, 212 waveforms were generated; as examples, the absolute and normalized waveforms of the three scenarios (preload, contractility, and afterload deficit) with a MAP of 60 mmHg are shown in Figure 2. The morphologies of the three reduced waveforms are clearly different from the control case, between the three underlying causes also morphological differences can be identified. Blood volume could be reduced from 6355 to 4355 ml (−32%) before the defined limits were met. Left ventricular contractility could be reduced from 2.62 to 0.42 mmHg/ml (−85%) and systemic vascular resistance from 1.09 to 0.22 mmHg*s/ml (−80%). Common clinical measures resulting from these simulations scenarios are displayed in Table 2.

**TABLE 2:** Clinically relevant measures from Aplysia simulations of hemodynamic instability in a 60‐year‐old adult

Clinical measure	Control	Preload BV reduction		Contractility LVC reduction	Afterload SVR reduction	
		−26%	−32%	−85%	−60%	−80%
SAP [mmHg]	140/74 (103)	75/47 (57)	60/40 (46)	72/50 (59)	94/40 (58)	72/26 (38)
PP [mmHg]	66	28	20	22	54	46
PAP [mmHg]	39/17 (23)	13/5 (7)	10/4 (5)	37/25 (28)	38/15 (21)	37/13 (20)
CVP [mmHg]	9	−1	−2	10	9	8
LAP [mmHg]	15	0	−1	24	12	9
RAP [mmHg]	9	−1	−2	10	9	8
CO [l/min]	5.46	2.93	2.28	2.52	6.22	6.69
SV [ml]	85	45	35	39	96	104
LVOT VTI [cm]	15.82	8.49	6.61	7.30	18.02	19.38
LVEF [%]	58	62	62	22	75	85
LV shorting fraction [%]	29	31	31	11	38	42
RVEF [%]	59	70	69	43	60	62
RV shorting fraction [%]	30	35	34	22	30	31
TAPSE [mm]	21	24	24	15	21	22

Abbreviations: BV, blood volume; CO, cardiac output; CVP, central venous pressure; LAP, left atrial pressure; LVOT VTI, left ventricular outflow tract velocity time integral; LVEF= left ventricular ejection fraction; LV, left ventricle; LVC, left ventricular contractility; PAP, pulmonary arterial pressure; PP, pulse pressure; RAP, right atrial pressure. CO; RV, right ventricle; RVEF, right ventricle ejection fraction; SAP, systolic arterial pressure; stroke volume, LVOT VTI; SVR, systemic vascular resistance; TAPSE, tricuspid annular plan systolic excursion.

**FIGURE 3:**
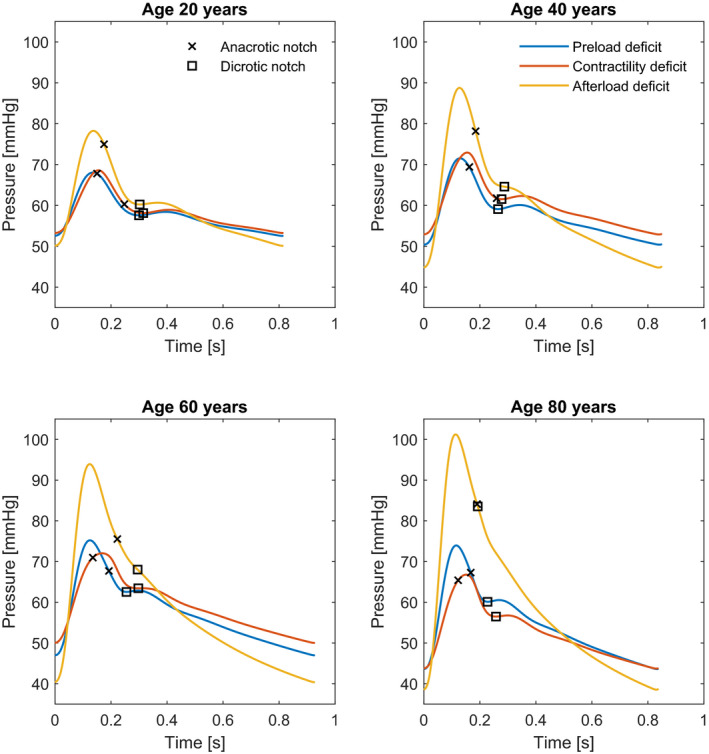
Arterial waveforms of simulated adults of 20, 40, 60, and 80 years old with reduced blood volume (blue), reduced left ventricular contractility (red) and reduced systemic vascular resistance (yellow). For these scenarios mean arterial pressure was aimed at 60 mmHg to make them comparable, the detected anacrotic and dicrotic notches are marked. The waveform morphologies of the three causes differ the most in the older adults. In the younger adults, the waveforms of the reduced preload and contractility look more alike; with reduced afterload the pulse pressure is clearly higher. For all ages, the dicrotic notch and timing of the systolic peak differ between the three causes

In Figure 3, the waveforms of adults with different ages are displayed, again with reduced BV, LVC, and SVR aimed at a MAP of 60 mmHg. The difference between the waveforms of the three underlying causes is clearer in the older adults, compared to the younger ones.

**FIGURE 4:**
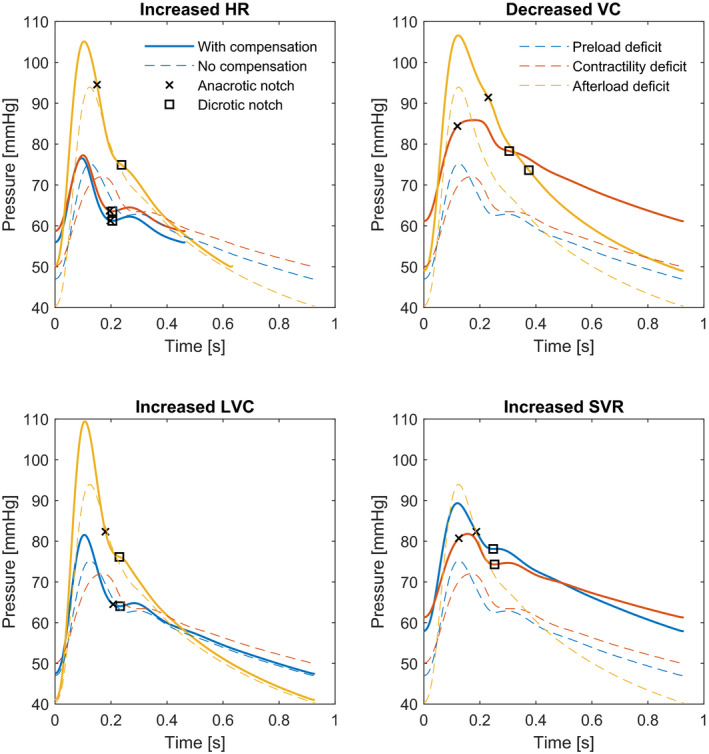
Arterial waveforms of a simulated 60‐year‐old adult with reduced blood volume (blue), reduced left ventricular contractility (red) and reduced systemic vascular resistance (yellow). The detected anacrotic and dicrotic notches are indicated with markers. In the reduced situations (dashed lines) the aim was to bring MAP to 60 mmHg to make the situations comparable. Baroreflex compensatory mechanisms were added until MAP was 70 mmHg (solid lines). To achieve this HR, LVC, and SVR were increased, and venous compliance decreased. For obvious reasons VC, LVC, and SVR were not changed in the situation of BV, LVC, and SVR reduction respectively. Heart rate influences the morphology of the waveform the most. Abbreviations: HR, heart rate; VC, venous compliance; LVC, left ventricular contractility; SVR, systemic vascular resistance; MAP, mean arterial pressure

The results of the addition of baroreflex compensatory mechanism to the 60‐year‐old adult case with MAP of 60 mmHg can be seen in Figure 4. Heart rate was increased to 95/min (+46%) in the reduced afterload scenarios to obtain a MAP of 70 mmHg. For the other two causes, HR was set at 130/min (+100%). The higher the HR the more the waveforms of the three scenarios resemble each other. Venous compliance was reduced to 88 ml/mmHg (−17%) in both causes. The increase of LVC had little effect on the mean arterial pressure; it was set at 5.22 mmHg/ml (+100%) for both causes. SVR was increased to 1.44 mmHg*s/ml (+32%) for the reduced preload scenario and to 1.82 mmHg*s/ml (+67%) for the contractility scenario. In general, the baroreflex compensatory mechanisms result in higher baseline and mean pressures, but the morphology of the waveforms was not changed considerably.

### 3.2 Sensitivity of waveform features

A selection of the results of the sensitivity analysis are displayed in Table 3, the results for all 45 features can be found in the Appendix A‐Table A2. A positive sensitivity value indicates that the waveform feature changes in the same direction as the input variable. None of the features show a strict linear relationship with the variables, although in the contractility scenarios the values of +10%, −10%, and −30% lie closer together. The features most sensitive to changes in BV, LVC, and SVR are: augmented pressure, augmentation index, and slope and duration of systolic upstroke and downstroke. Features often change in opposite direction depending on the changed variable, making them suitable for differentiation between scenarios (Table 3).

**TABLE 3:** Results of the sensitivity analysis

Variable Arterial waveform feature	Preload BV change			Contractility LVC change			Afterload SVR change		
	+10%	−10%	−30%	+10%	−10%	−30%	+10%	−10%	−30%
Augmented pressure	−45.65	−13.99	−3.90	2.82	1.48	1.54	−18.58	−6.41	−9.37
Augmentation index	−39.84	−17.22	−18.31	2.30	1.08	1.21	−18.34	−6.74	−10.47
Systolic downstroke pressure	1.28	−0.33	1.30	0.55	0.48	0.57	−0.48	−0.82	−1.09
Descending index	−0.54	−1.72	−2.73	0.12	0.02	0.07	−0.74	−1.02	−1.48
Dicrotic notch index	0.22	0.69	1.09	−0.05	−0.01	−0.03	0.30	0.41	0.59
Characteristic impedance	3.72	−0.61	−1.35	−0.03	−0.17	−0.29	0.31	0.05	−0.55
dP/dt max	1.21	1.17	2.27	0.63	0.68	0.72	0.18	0.17	0.26
Slope systolic upstroke	−1.93	0.48	2.14	0.77	0.77	0.83	−2.57	0.03	0.09
Slope systolic downstroke	9.55	0.65	0.89	0.28	0.22	0.20	4.56	−0.21	−0.28
Duration systolic upstroke	4.78	0.74	0.18	−0.32	−0.32	−0.42	3.85	0.14	0.18
Duration systolic downstroke	−4.21	−1.05	0.55	0.26	0.26	0.40	−3.45	−0.59	−0.75
Myocardial oxygen ratio	0.25	0.25	−1.20	−0.09	−0.07	−0.15	0.35	0.46	0.56
Relative myocardial oxygen ratio	0.24	0.45	−0.98	−0.11	−0.03	−0.18	0.52	0.62	0.78
Stroke volume	0.07	0.16	1.06	0.21	0.23	0.26	−0.22	−0.25	−0.38
Cardiac output	0.07	0.16	1.06	0.21	0.23	0.26	−0.22	−0.25	−0.38

Note

One‐at‐a‐time sensitivity analysis for changes in preload, contractility, and afterload. The ratio between the percentual change of variable and percentual change of the arterial waveform feature are displayed. Only the features which show a clear difference in sensitivity for the three scenarios are shown here.

Abbreviations: BV, blood volume; LVC, left ventricular contractility; SVR, systemic vascular resistance.

Respiratory variation did not significantly affect the waveform features. The relative standard deviation of the features during 60 s of spontaneous breathing did not exceed 5% of the mean.

### 3.3 Principal components

**FIGURE 5:**
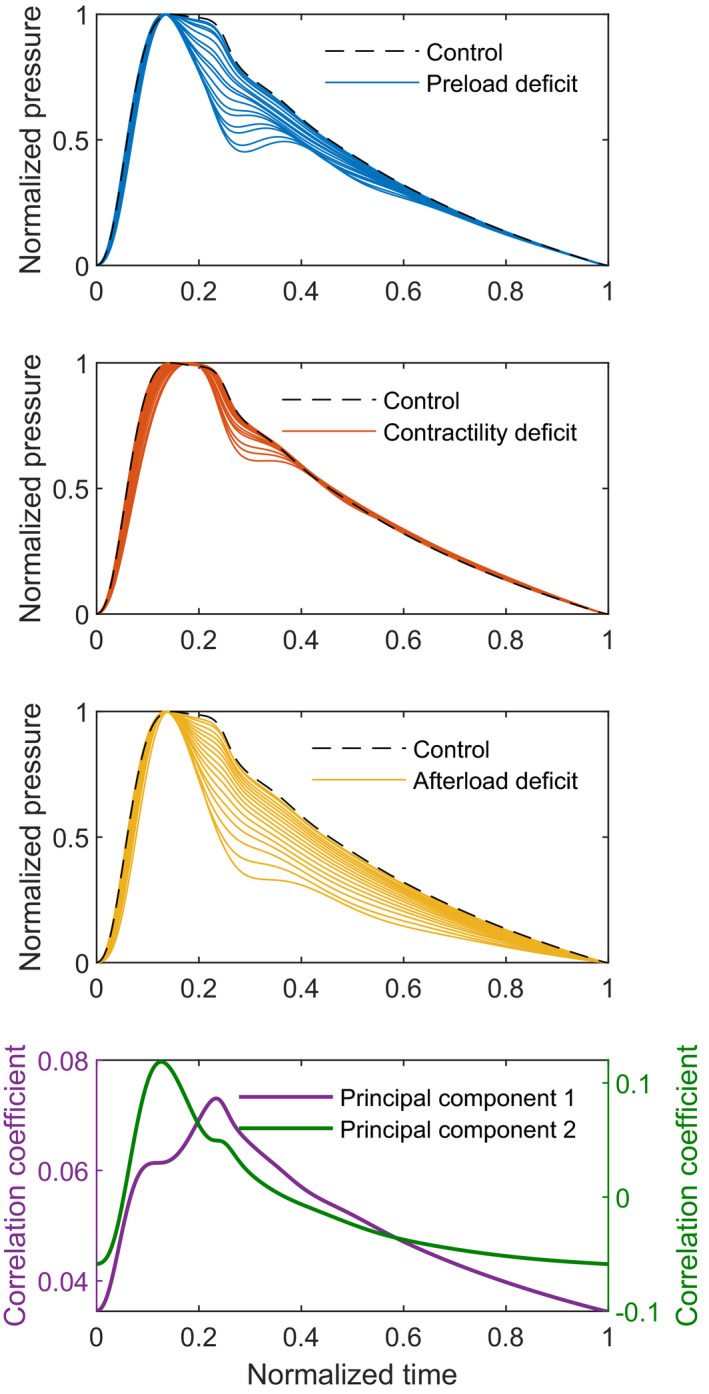
Arterial waveforms of a simulated 60‐year‐old adult (top three subplots) with normal physiology (black), reduced blood volume (blue, steps of 2%), reduced left ventricular contractility (red, steps of 5%) and reduced systemic vascular resistance (yellow, steps of 5%). All waveforms are normalized, ranging from 0 to 1 in both pressure and time. The most variation can be seen in the systolic downstroke and dicrotic notch area. The correlation coefficients (bottom) of the first (purple) and second (green) principal components, show a high correlation at the systolic peak, upstroke, downstroke and dicrotic notch

The first PCA was performed only on the waveforms of the control 60‐year‐old adult, with stepwise reduction of BV, LVC, and SVR. The PCA of the raw signals resulted in a first principal component (PC) explaining 97.5% and a second PC explaining 2.4% of the variation between the arterial waveforms. The correlation coefficient of the first PC is the highest in the systolic downstroke and the second PC is maximal during the systolic upstroke and systolic peak pressure (Figure 5, bottom). The waveforms (Figure 5, top) also show the most variability in this timeframe.

**FIGURE 6:**
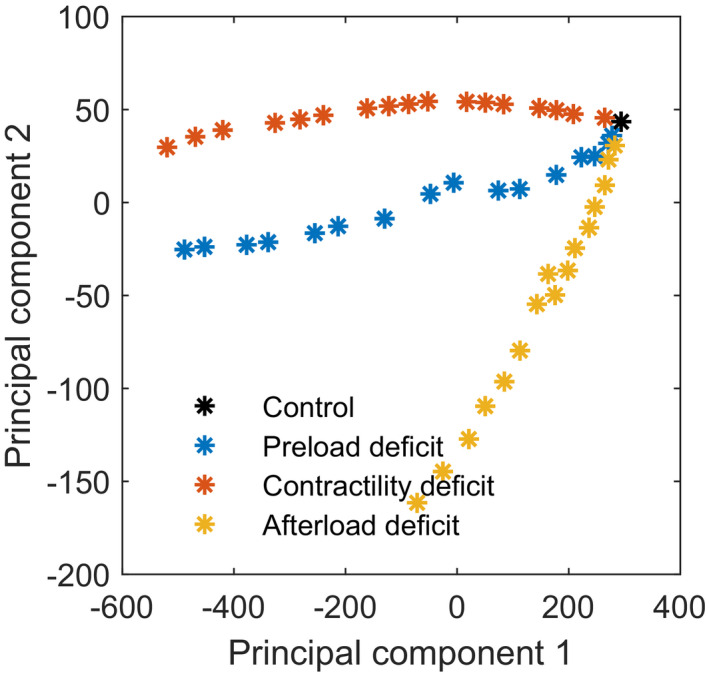
Scores of the first two principal components based on arterial waveform features of a 60‐year‐old adult with normal physiology (black), reduced blood volume (blue, steps of 2%), reduced left ventricular contractility (red, steps of 5%), and reduced systemic vascular resistance (yellow, steps of 5%). Based on these principal components a clear distinction can be made between the three scenarios

In Figure 6, the results of the PCA of the calculated arterial waveform features are depicted. The different causes of HD instability can easily be distinguished and can be described by a linear or slightly curved trend. The first PC explains 94.4% and the second PC explains 4.8% of the variability between the features. The features with a correlation coefficient for the first PC above 0.25 are dP/dt max and slope systolic upstroke. For the second PC these are: slope of the systolic downstroke, anacrotic notch pressure, dicrotic notch pressure, and diastolic peak pressure.

**FIGURE 7:**
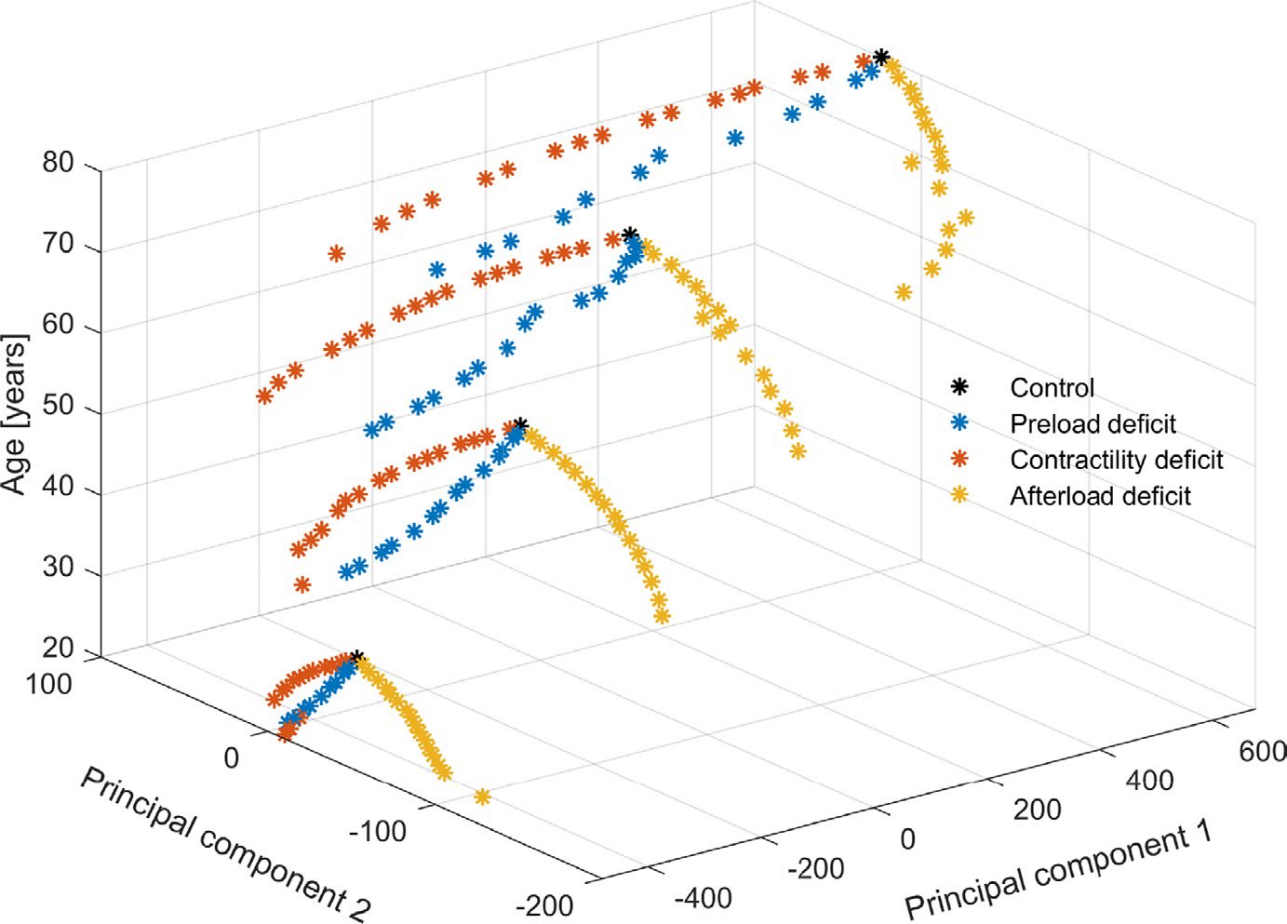
Scores of the first two principal components based on the arterial waveform features of four simulated adults: 20, 40, 60, and 80 years old. In each of the cases normal physiology (black) is shown, together with reduced blood volume (blue, steps of 2%), reduced left ventricular contractility (red, steps of 5%), and reduced systemic vascular resistance (yellow, steps of 5%). Within one age a distinction can be made between the three reduction scenarios, but between cases of different ages this is not possible

The results of the PCA using the waveform features of all four adult cases with different ages are displayed in Figure 7. The overall explanation is 96.1% for the first PC and 2.9% for the second PC. The features with a correlation coefficient of the PC1 higher than 0.25 are again dP/dt max and slope of the systolic upstroke. For PC2 anacrotic notch, dicrotic notch, diastolic peak, mean arterial and systolic peak pressure, and stroke volume and slope of the systolic downstroke are highly correlated. Within each case, the three scenarios can be distinguished from each other using these PCs. However, a comparison between cases of different age is not possible, since the PCs overlap.

## 4 DISCUSSION

This simulation study shows that a differentiation can be made between underlying causes of HD instability based on the arterial waveform morphology and derived features. Deficits in preload, contractility, and afterload can be distinguished from each other in different individuals and when compensatory baroreflex mechanisms are simulated. The most discriminating parts of the arterial waveform are the systolic upstroke and downstroke, and the anacrotic and dicrotic notch.

### 4.1 Physiological mechanisms

A more in‐depth analysis of the arterial waveform is provided in this paragraph, to further explain the morphological changes during HD instability.

The PCA reveals that the slope of the systolic upstroke, that is, dP/dt max and average slope, varies the most during HD instability, as reflected by the close correlation with the first principal component. When pre‐ and afterload are kept constant, the intrinsic left ventricular contractility and the slope of the systolic upstroke are linearly related, since this part of the waveform is strongly influenced by cardiac ejection. In general, it remains a matter of debate, whether peripherally measured arterial dP/dt max sufficiently reflects left ventricular dP/dt max, the latter being classically accepted as a measure of cardiac contractility (Monge Garcia et al., 2018; Tartiere et al., 2007; Vaquer et al., 2019). Data from other clinical studies suggest that abrupt changes in dP/dt max accurately reflect changes in LV contractility, but only when patients are adequately filled (De Hert et al., 2006; Morimont et al., 2012; Scolletta et al., 2013). Concluding, the systolic upstroke can independently or in combination be influenced by all the different primary causes of HD instability, that is, preload, contractility, and afterload, and does therefore not allow to sufficiently differentiate between them.

The systolic downstroke, and anacrotic and dicrotic notches are correlated to the second principal component and therefore allow further discrimination of the pathophysiological origin of HD instability. The distinct morphology of these parts of the wave can be explained by wave reflection mechanisms, which are made visible by the separation of the forward wave and reflected wave. In the scenario of reduced afterload, a high, broad, and steep forward wave is observed and a small, reflected wave, leading to a steep systolic downstroke (Appendix A‐Figure A1). These findings are in accordance with the general notion that a sharp downstroke is indicative of a reduced resistance to blood flow, implying vasodilation, (Esper & Pinsky, 2014) although resistance is not the only determinant of wave reflection (Westerhof & Westerhof, 2012).

In addition, the slope of the systolic downstroke is also influenced by the appearance of a C‐type anacrotic notch, see also Figure 1. Although analysis of the anacrotic notch, and the derived augmentation index, is currently only used in hypertension research, they can be accurately measured in low blood pressure states as well (Papaioannou et al., 2004). The data show C‐type anacrotic notches in most peripherally measured waveforms, while in the scenario of reduced contractility an A‐type notch in the systolic upstroke was found (Figure 2 and Table 3). This A‐type notch becomes visible due to a reduced and prolonged cardiac ejection, which results in a broad and later peak of the forward wave (Appendix A‐Figure A1).

Similar to the anacrotic notch, the peripheral dicrotic notch is also a result of wave reflection and it is more prominent when both the forward and reflected waves become separated in time. This can clearly be seen in scenarios of reduced preload and contractility, by a lower dicrotic notch pressure and a higher diastolic peak (Figure 2 and Table 3). These findings are consistent with previous research stating that the dicrotic notch decreases with hypovolemia (Convertino et al., 2016; Wasicek et al., 2021) and even describing a negative relative notch pressure, that is, a dicrotic notch below the diastolic baseline (Hsieh & Hung, 2016). The systolic‐dicrotic notch pressure difference, a description of the systolic downstroke, is also investigated in clinical studies and associated with fluid responsiveness and myocardial contractility (Messina et al., 2021; Morelli et al., 2020). On the contrary, in reduced afterload the forward wave is relatively large and eclipses the reflected wave, so that the dicrotic notch is not visible (Appendix A‐Figure A1). Clinical data support these findings, as a depression of the dicrotic notch is seen in vasodilated states and increase of the dicrotic notch pressure with vasoconstrictive medication (Bhagat et al., 2011; Politi et al., 2016).

To summarize, the morphological changes of the arterial waveform have a plausible (patho)physiological underpinning. In addition, it can be inferred from the sensitivity analysis that several waveform features are sensitive for hemodynamic instability, with reducing blood volume having the greatest impact on the waveform. Even with the imposed respiratory variation of spontaneous breathing, the waveform features of a single heartbeat are sufficiently stable as based on the proposed analysis. While other experimental studies only investigated a single facet of HD instability, data from this study allow to identify those features discriminating between preload, contractility, and afterload deficiency. As clearly reflected in the PCA, a combination of the features with the highest correlation coefficients enable the differentiation between underlying causes. Importantly, the more severe the HD instability, the more the principal components diverge from each other, thus the clearer the differentiation.

Our novel approach of peripheral arterial waveform analysis allows to discriminate underlying causes of HD instability within one simulated individual, but is limited when comparing individuals. PCs of simulations of varying ages are not unique for elements of HD instability in different individuals. In addition, differentiation between the three primary causes of HD instability is less clear in young patients, since they have more compliant vessels and therefore less wave reflection. This is also noted by the results from the Framingham heart study, showing a more prevalent dicrotic notch in younger individuals (Bhagat et al., 2011). From these findings it can be concluded that following trends of waveform features over time within an individual, especially in the elderly, is the most feasible opportunity for improved monitoring of HD changes using our proposed analysis.

Under clinical circumstances of hemodynamic instability also baroreflex compensation mechanisms come into play, leading to sympathetic activation, which influences the arterial pressure waveform. In the simulations with increased heart rate, the shorter systole becomes a more prominent factor defining waveform morphology. Therefore, the waveforms of the three scenarios resemble each other, but still exhibit a delayed systolic peak with a contractility deficit and a higher dicrotic notch with the afterload deficit. Other sympathetic compensatory mechanisms, being SVR increase and VC decrease, only influence MAP, but do not change the waveform morphology. When LVC is increased, the systolic upstroke and systolic peak increase, but this does not conflict with the differentiation between the underlying causes, since that is mostly based on the results of wave reflection. In summary, adding baroreflex compensation results in more similar waveform morphology of the three scenarios, but the important identifying characteristics remain present.

### 4.2 Methodological advantages and limitations

The advantage of an in‐silico simulation study is that all variables and possible confounders are strictly controlled. This allows in‐depth understanding of the mechanisms behind arterial waveform morphology and helps to generate hypotheses for further clinical studies.

The adjustments made to the input variables to obtain HD unstable scenarios were comparable with clinical values from the literature, suggesting that the simulations produce realistic scenarios for HD instability and circulatory shock. In the simulation, a maximum BV reduction of 32% was reached, comparable with the 30% of total blood volume loss reported as a threshold for hypotensive shock due to hemorrhage (Bonanno, 2020). SVR was decreased till 0.22 mmHg*s/ml, which is much in line with the reported 0.33 mmHg*s/ml in septic patients (Melo & Peters, 1999). A 75% reduction in cardiac contractility reported in cardiogenic shock (Bleifeld et al., 1974) is comparable to the 85% decrease in this study. As a consequence, the left ventricular ejection fraction (LVEF) decreases from 58% to a minimum of 23% (Table 2). This matches the ranges of normal (LVEF >55%) and severely abnormal (<30%) as based on current echocardiography guidelines (Lang et al., 2015).

Despite the realistically chosen values of the input variables, the simulation scenarios are inherently limited to a certain extent. This study focused on the separate decrease of BV, LVC, and SVR, but other pathology causing hemodynamic instability could also be present (e.g., right ventricular failure, diastolic failure or a significantly altered right‐left interdependence). Besides, in septic shock a complex combination of pathophysiological mechanisms is often present, causing a dynamic combination of relative hypovolemia, vasoplegia, and septic cardiomyopathy. Nevertheless, when the effect of distinct single causes of hemodynamic instability on the arterial waveform is well known, the contribution of these pathological elements to complex real‐life cases could more easily be unraveled.

The Aplysia CardioVascular Lab was used in this study, which realistically simulates peripheral pressure waveforms, though there are some inherent shortcomings. It is a comprehensive and complex model of the human cardiovascular system, therefore, it is virtually impossible to determine all its parameters with clinically available measurements. Several assumptions, model simplifications, and non‐modelled parameters are used, so Aplysia only approaches real human hemodynamics. However, some specific (patho)physiological states were validated using clinical and experimental data, showing comparable results (Broomé et al., 2013; Broomé & Donker, 2016; Lindfors et al., 2017; Maksuti et al., 2019). The peripheral pressure and flow are not specifically validated, but appear realistic and comparable with earlier research on arterial waveforms (Remington & Wood, 1956; Mills et al., 1970; Murgo et al., 1980; Shah & Bedford, 2001).

### 4.3 Clinical perspectives

There is a demand for more personalized and pathophysiology based medicine in the ICU (Vincent et al., 2015). Arterial waveform analysis can contribute to this need in several ways. By discriminating between the three elementary deficits underlying unstable hemodynamics based on a model‐based method as outlined in this study, a more well‐informed HD management strategy can be pursued. Administration of fluids, vasopressive, and/or inotropic medication can be tailored to the patient's needs and their effect can closely be monitored by a relatively simple analysis of arterial line tracings.

In combination with focused bedside echocardiography, arterial waveform analysis can be a powerful tool to continuously assess ventriculo‐arterial coupling. Additionally, specific waveform features can be used to set individual hemodynamic targets in conjunction with information on, for example, right ventricular function and interventricular dependency as readily assessable by echocardiography.

Importantly, the algorithms used in this study include well‐defined, straightforward analyses and could thus possibly be automated and included into existing modern monitoring devices, including HD monitors and echocardiography devices. Such a comprehensive HD monitoring strategy may lead to a more insightful, patient‐specific management and it is tempting to speculate whether this approach will also translate into an improvement of clinical outcome measures related to the ICU stay.

## 5 CONCLUSIONS

The proposed approach allows identifying distinct peripheral arterial waveform morphologies and differentiate between preload, contractility, and afterload deficits as causes of hemodynamic instability. These findings set the stage for improved, individualized strategies towards easily‐accessible, continuous, and comprehensive hemodynamic monitoring at the bedside. Further experimental and clinical research is warranted to advance the proposed algorithms and ultimately prove their usability in clinical practice to optimize daily management in critically ill patients.

## CONFLICT OF INTEREST

MB is the founder and owner of the company Aplysia Medical AB, Stockholm, Sweden, having the commercial rights of the simulation model and software used in this work. The other authors have no conflicts of interest to declare.

## AUTHOR CONTRIBUTIONS

MM and BW conceived the idea and designed the study. MB developed and improved the model. MM performed the simulations, analyzed the data, and wrote the manuscript. All authors discussed the results and drafted, reviewed and accepted the manuscript.

## Figures and Tables

**FIGURE A1:**
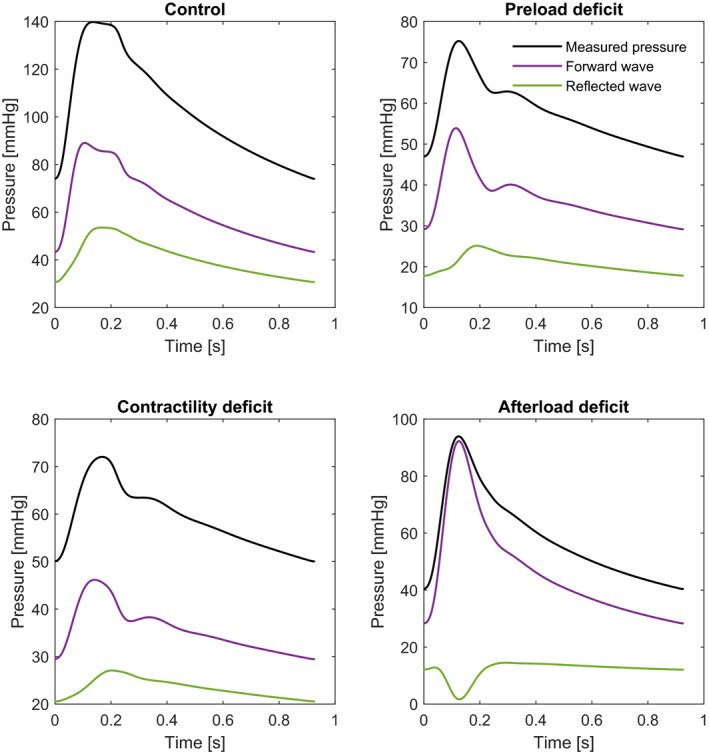
Results of wave separation in a 60‐year‐old control subject and with hemodynamic instability based on reduced preload, contractility, and afterload with a mean arterial pressure at 60 mmHg

## APPENDIX A

**TABLE A1:** Arterial waveform features

Feature	Abbreviation	Unit	Definition
Absolute pressures			
Systolic blood pressure	SBP	mmHg	max (p)
Diastolic blood pressure	DBP	mmHg	min (p)
Mean arterial pressure	MAP	mmHg	mean (p)
Dicrotic notch pressure	DNP	mmHg	p @ dicrotic notch
Anacrotic notch pressure	ANP	mmHg	p @ anacrotic notch
Diastolic peak pressure	DPP	mmHg	max (p (diastole))
Forward peak pressure	FPP	mmHg	max (p_fwd)
Reflected peak pressure	RPP	mmHg	max (p_refl)
Relative pressures			
Pulse pressure	PP	mmHg	SBP ‐ DBP
Relative dicrotic notch pressure	rDNP	mmHg	DNP ‐ DBP
Dicrotic notch index	DNIx	%	(rDNP / PP) * 100
Descending pressure	DP	mmHg	SBP ‐ DNP
Descending index	DIx	%	(DP / PP) * 100
Diastolic upswing pressure	DUSP	mmHg	DPP ‐ DNP
Upswing index	USIx	%	(DUPS / PP) * 100
Augmentation pressure	AP	mmHg	SBP – ANP *if A*‐*type notch* ANP – SBP *if C*‐*type notch*
Augmentation index	AIx	%	(AP / PP) * 100
Reflection pressure ratio	RPratio	–	RPP / FPP
Durations			
Duration heartbeat	T_beat	s	t @ end
Heart rate	HR	/min	60 / HR
Duration systole	T_sys	s	t @ dicrotic notch
Duration upstroke systole	T_upsys	s	t @ systolic peak
Duration downstroke systole	T_downsys	s	T_sys – T_upsys
Duration diastole	T_dia	s	T_beat – T_sys
Time to forward peak	T_fwd	s	t @ forward peak
Time to reflected peak	T_refl	s	t @ reflected peak
Reflection time ratio	T_reflratio	–	T_refl / T_fwd
Slopes			
Systolic upstroke slope	S_upsys	mmHg/s	(SBP ‐ DBP) / T_upsys
Systolic downstroke slope	S_downsys	mmHg/s	(DNP ‐ SBP) / T_downsys
Diastolic runoff slope	S_dia	mmHg/s	(DBP ‐ DPP) / T_dia
Maximum slope	dP/dt_max_	mmHg/s	max (p’)
Areas			
Total area	A_beat	mmHg*s	∫p
Relative total area	rA_beat	mmHg*s	A_beat – (DBP_beat)
Systolic area	A_sys	mmHg*s	∫p(systole)
Relative systolic area	rA_sys	mmHg*s	A_sys – (DBP T_sys)
Diastolic area	A_dia	mmHg*s	∫p(diastole)
Relative diastolic area	rA_dia	mmHg*s	A_dia – (DBP T_dia)
Myocardial oxygen supply demand ratio	O2ratio	‐	A_dia / A_sys
Relative myocardial oxygen supply demand ratio	rO2ratio	‐	rA_dia / rA_sys
Forward wave area	A_fwd	mmHg*s	$\int p_{\mathrm{fwd}}$
Reflected wave area	A_refl	mmHg*s	$\int p_{\mathrm{refl}}$
Reflection area ratio	A_reflratio	‐	A_refl / A_fwd
Other			
Stroke volume	SV	ml	1/3.5 (PP / (SBP + DBP)) * 1000
Cardiac output	CO	l/min	(SV / 1000) * HR
Characteristic impedance	Z0	Ω	Z = fft (p) / fft (q) Z0 = mean (abs (Z (5:15)))

Abbreviations: fft, fast fourier transform; p, arterial pressure signal; p_fwd, forward wave pressure; p_refl, reflected wave pressure; p’, first derivative pressure signal; q, flow signalt, time signal.

**TABLE A2:** Results sensitivity analysis

Variable Arterial waveform feature	Preload BV change:			Contractility LVC change:			Afterload SVR change:		
	+10%	−10%	−30%	+10%	−10%	−30%	+10%	−10%	−30%
Systolic blood pressure	1.86	1.07	1.81	0.26	0.29	0.33	0.46	0.36	0.51
Diastolic blood pressure	1.81	0.97	1.45	0.11	0.14	0.17	0.62	0.52	0.72
Mean arterial pressure	1.79	1.12	1.72	0.19	0.22	0.26	0.56	0.47	0.67
Dicrotic notch pressure	1.95	1.29	1.89	0.21	0.26	0.29	0.61	0.54	0.75
Anacrotic notch pressure	1.43	1.35	1.91	0.21	0.27	0.31	0.49	0.48	0.69
Diastolic peak pressure	1.95	1.29	1.87	0.21	0.26	0.29	0.61	0.54	0.75
Forward peak pressure	2.15	0.74	1.63	0.28	0.28	0.31	0.22	0.14	0.03
Reflected peak pressure	0.78	1.39	2.00	0.24	0.31	0.38	0.61	0.58	1.05
Pulse pressure	1.93	1.18	2.21	0.42	0.46	0.52	0.29	0.18	0.26
Relative dicrotic notch pressure	2.19	1.79	2.57	0.37	0.46	0.49	0.59	0.58	0.81
Dicrotic notch index	0.22	0.69	1.09	−0.05	−0.01	−0.03	0.30	0.41	0.59
Systolic downstroke pressure	1.28	−0.33	1.30	0.55	0.48	0.57	−0.48	−0.82	−1.09
Descending index	−0.54	−1.72	−2.73	0.12	0.02	0.07	−0.74	−1.02	−1.48
Augmentation pressure	−45.65	−13.99	−3.90	2.82	1.48	1.54	−18.58	−6.41	−9.37
Augmentation index	−39.84	−17.22	−18.31	2.30	1.08	1.21	−18.34	−6.74	−10.47
Reflection pressure ratio	−1.12	0.69	0.73	−0.04	0.03	0.08	0.38	0.46	1.03
Duration heartbeat	0.00	0.00	0.00	0.00	0.00	0.00	0.00	0.00	0.00
Heart rate	0.00	0.00	0.00	0.00	0.00	0.00	0.00	0.00	0.00
Duration systole	−0.17	−0.25	0.39	0.00	0.00	0.03	−0.17	−0.26	−0.33
Duration upstroke systole	4.78	0.74	0.18	−0.32	−0.32	−0.42	3.85	0.14	0.18
Duration downstroke systole	−4.21	−1.05	0.55	0.26	0.26	0.40	−3.45	−0.59	−0.75
Duration diastole	0.08	0.12	−0.18	0.00	0.00	−0.01	0.08	0.12	0.16
Time to forward peak	8.51	0.00	−0.47	0.00	−0.21	−0.23	0.00	−0.19	−0.24
Time to reflected peak	2.52	0.30	−0.64	−0.39	−0.26	−0.29	0.89	−0.12	−0.49
Reflection time ratio	−3.23	0.30	−0.15	−0.39	−0.05	−0.06	0.89	0.07	−0.24
Systolic upstroke slope	−1.93	0.48	2.14	0.77	0.77	0.83	−2.57	0.03	0.09
Systolic downstroke slope	9.55	0.65	0.89	0.28	0.22	0.20	4.56	−0.21	−0.28
Diastolic runoff slope	2.09	1.69	2.56	0.37	0.46	0.51	0.51	0.46	0.68
Maximum slope (dP/dt max)	1.21	1.17	2.27	0.63	0.68	0.72	0.18	0.17	0.26
Total area	1.79	1.12	1.72	0.19	0.22	0.26	0.56	0.47	0.67
Relative total area	1.74	1.51	2.42	0.39	0.45	0.52	0.41	0.36	0.54
Systolic area	1.61	0.98	2.01	0.24	0.27	0.35	0.33	0.20	0.36
Relative systolic area	1.61	1.33	2.52	0.44	0.46	0.58	0.17	0.09	0.22
Diastolic area	1.90	1.21	1.54	0.15	0.20	0.21	0.70	0.65	0.86
Relative diastolic area	1.89	1.72	2.29	0.32	0.43	0.43	0.70	0.71	0.94
Myocardial oxygen supply demand ratio	0.25	0.25	−1.20	−0.09	−0.07	−0.15	0.35	0.46	0.56
Relative myocardial oxygen supply demand ratio	0.24	0.45	−0.98	−0.11	−0.03	−0.18	0.52	0.62	0.78
Forward wave area	2.25	0.97	1.64	0.19	0.21	0.23	0.49	0.38	0.44
Reflected wave area	1.07	1.35	1.84	0.18	0.25	0.31	0.66	0.62	1.03
Reflection area ratio	−0.96	0.42	0.39	−0.01	0.04	0.08	0.17	0.25	0.68
Stroke volume	0.07	0.16	1.06	0.21	0.23	0.26	−0.22	−0.25	−0.38
Cardiac output	0.07	0.16	1.06	0.21	0.23	0.26	−0.22	−0.25	−0.38
Characteristic impedance	3.72	−0.61	−1.35	−0.03	−0.17	−0.29	0.31	0.05	−0.55

Abbreviations: BV, blood volume; LVC, left ventricular contractility; SVR, systemic vascular resistance.
